# Treatment outcomes, antibiotic use and its resistance pattern among neonatal sepsis patients attending Bahawal Victoria Hospital, Pakistan

**DOI:** 10.1371/journal.pone.0244866

**Published:** 2021-01-13

**Authors:** Muhammad Atif, Rabia Zia, Iram Malik, Nafees Ahmad, Sajjad Sarwar

**Affiliations:** 1 Department of Pharmacy Practice, Faculty of Pharmacy, The Islamia University of Bahawalpur, Bahawalpur, Punjab, Pakistan; 2 Department of Pharmacy Practice, Faculty of Pharmacy and Health Sciences, University of Balochistan, Quetta, Balochistan, Pakistan; 3 Department of Pulmonology, Bahawal Victoria Hospital, Bahawalpur, Punjab, Pakistan; Nitte University, INDIA

## Abstract

**Background:**

Sepsis is one of the major causes of neonatal mortality in Pakistan. This study aimed to investigate the treatment outcomes, antibiotic use and its resistance pattern among neonatal sepsis patients attending a tertiary care hospital in Pakistan. We also aimed to identify the factors affecting mortality in neonatal sepsis patients.

**Methods:**

A descriptive, cross-sectional study was conducted in the pediatric wards of the Bahawal Victoria Hospital, Bahawalpur, Pakistan. All eligible neonatal sepsis patients who were registered at the study site from January 1, 2019 to June 30, 2019 were included in the study. The data collection form included information on patient’s characteristics, antibiotic use and its sensitivity pattern, laboratory and microbiological data, and final treatment outcomes. Treatment outcomes included, discharged (with treatment success), leave against medical advice (LAMA), discharged on request (DOR) and death. Multivariable binary logistic regression analysis was used to find the independent factors associated with death. A p-value of less than 0.05 was considered statistically significant.

**Results:**

Among the total 586 patients, 398 (67.9%) were male, 328 (56%) were preterm, 415 (70.8%) were diagnosed with early onset sepsis, 299 (51%) were born with low birth weight. Most of the patients (n = 484, 82.6%) were treated with amikacin+cefotaxime at the start of treatment. Culture was positive in 52 (8.9%) patients and the most commonly identified bacteria included, *Klebsiella* species (n = 19, 36.5%) followed by *E*. *coli* (n = 15, 28.5%) and *Staphylococcus aureus* (n = 8, 15.4%). The identified bacterial isolates showed high level of resistance against the antibiotics initiated at the start of the treatment, while resistance against piperacillin+tazobactam, imipenem, vancomycin and linezolid was very low. Just under half of the patients (n = 280, 47.8%) successfully completed the treatment (i.e., discharged with treatment success), while 123 (21%) patients died during treatment. In multivariable binary logistic regression, the factors which still remained significantly associated with neonatal death included, preterm delivery (AOR 9.59; 95% CI 4.41, 20.84), sub-optimal birth weight (AOR 5.13; 95% CI 2.19, 12.04), early onset sepsis (AOR 2.99; 95% CI 1.39, 6.41) and length of hospital stay (AOR 0.76; 95% CI 0.67, 0.88).

**Conclusion:**

The mortality rate associated with sepsis was high in our study cohort. The bacterial isolates showed high level of resistance against the antibiotics started as the empiric therapy. Rational use of antibiotics can decrease the adverse outcomes in neonatal sepsis patients.

## Introduction

Neonatal sepsis is defined as a systemic infection which occurs before 28 days of newborn’s life [1]. It is manifested by hemodynamic, post-inflammatory and immunosuppressive changes that can lead to substantial mortality and morbidity [2]. The clinical signs and symptoms of neonatal sepsis include, hypothermia or fever, respiratory problems such as apnea and cyanosis, difficulty in feeding, abdominal distension, diarrhea, vomiting, oliguria, lethargy and irritability [2]. On the basis of onset of symptoms, neonatal sepsis could be either early-onset sepsis (EOS) or late-onset sepsis (LOS). The EOS manifests itself within first 72 hours and is vertically transmitted [3]. Whereas, the LOS manifests itself after 72 hours of child birth and is mainly acquired horizontally from the environment [4].

Neonatal sepsis is the leading cause of mortality worldwide, but compared with high income countries, its prevalence and mortality rates are high in low and middle-income countries (LMICs) probably due to poor hygiene and suboptimal practices for infection control [5]. The neonatal mortality rate (per 1000 live births) in Pakistan has declined from 55 in 2013 to 42 in 2018 [6, 7], however, it is much higher compared with the developed countries like the United States (US), the United Kingdom (UK) and Canada where it is less than 5 per 1000 live births. The neonatal mortality rate in Pakistan is even much higher than its neighboring countries, for example, India (23 per 1000 live births), Iran (9 per 1000 live births) and China (4 per 1000 live births) [8]. Compared with the global scenario, Pakistan appears to be lagging behind in achieving the target set (12 or fewer neonatal deaths by 2030) by the Every Newborn Action Plan (ENAP) [9]. Among the known causes of neonatal mortality in Pakistan, sepsis accounted for 17.2% of the total deaths (2015 data) [10].

The type of pathogens causing neonatal sepsis differ among countries, however, literature from developing countries suggest that although gram-negative organisms predominate, but some of the gram-positive bacteria such as *Staphylococcus aureus* and *coagulase negative staphylococcus* (CoNS) may also cause neonatal sepsis [11–14]. The World Health Organization (WHO) has recommended to use injectable gentamicin and ampicillin as a first-line therapy for the hospitalized neonatal sepsis patient, and according to a recent review, there are no evidences which suggest to change this therapy [15]. However, due to emergence of antimicrobial resistance and sepsis associated complications, use of many other antibiotics (either alone or in combination) such as cephalosporins, imipenem, vancomycin, piperacillin/tazobactam (PT), amikacin, metronidazole and clindamycin has been recommended in many current international guidelines [15].

Antibiotic resistance is a growing problem worldwide and poses a threat to survival rates from serious infections [16], including neonatal sepsis. Globally, about 214,000 neonates die each year owing to sepsis caused by bacteria resistant to first-line antibiotics [17]. Though there is no availability of data on this aspect from Pakistan, but seriousness of the issue can be gauged by prevailing inappropriate use of antibiotics among pediatrics, availability of inappropriate antibiotic pack sizes for pediatrics and poor adherence to treatment guidelines [18, 19]. Most importantly, there is a variation in the pathogen profile and their susceptibility pattern against antimicrobials among various countries, and it changes over time [14]. According to an estimate, a total of 25,692 neonates in Pakistan succumb to resistant sepsis infections each year [17]. In this regard, it is important to know the bacterial isolates and their resistance pattern against commonly used antibiotics in neonatal sepsis not only at a country level but also at all levels of healthcare [3]. Based on the fact that neonatal mortality rate is very high in Pakistan [20] and sepsis is one of the major causes of neonatal mortality [6, 10], such evidence based data will help the clinicians to initiate an empiric therapy which can decrease the probability of adverse outcomes among the patients. Despite the gravity of matter, only a few studies were conducted in Pakistan among neonatal sepsis patients [11, 21, 22], however, antibiotic use pattern and final treatment outcomes have not been discussed in detail. Moreover, patient’s attributes associated with the final treatment outcome are still under explored in Pakistan. Therefore, the aim of this study was to investigate the treatment outcomes, antibiotic use and its resistance pattern among neonatal sepsis patients in Pakistan. We also identified the independent factors associated neonatal mortality in our study cohort.

## Methodology

### Study setting

This study was conducted in the pediatric wards of the Bahawal Victoria Hospital (BVH), Bahawalpur, Punjab, Pakistan. The BVH has a bed capacity of 1600, and it serves large population living in the southern part of the province of Punjab [23]. The hospital has more than 20 departments including, medicine, surgery, pediatrics, radiology, pulmonology, obstetrics and gynecology, pathology, dermatology, cardiology, neurology, nephrology, physiotherapy, endocrinology, and allied medicine. The BVH has two pediatric wards named as Pediatric Ward 1 and Pediatric Ward 2 with the respective bed capacity of 94 and 82, respectively.

At the study setting, neonatal sepsis is diagnosed on the basis of clinical signs and symptoms (i.e., temperature instability, feeding problems, convulsions, lethargy and respiratory distress) [13], laboratory findings such as C-Reactive Protein (CRP), complete blood count (CBC) (clinical sepsis) and/or blood/urine cultures [12, 24]. Given the extremely high burden on microbiological laboratory at the BVH, all presumptive cases of neonatal sepsis are not necessarily diagnosed through positive cultures. At the time of admission in the pediatrics wards, neonatal sepsis patients (diagnosed clinically) are put on empiric parenteral antibiotics. However, the doctors recommend to do cultures in patients who do not show clinical improvement. On an average, cultures are ordered after two days of initiating empiric therapy.

### Study design and study population

This was a descriptive, cross-sectional study. All neonates admitted in the Pediatric Ward 1 and Pediatric Ward 2 from January 1, 2019 to June 30, 2019, and diagnosed with EOS and LOS were included in the study. Neonates with extremely low birth weight (i.e., <1000 g) and those suffering from pneumonia, meningitis and congenital abnormalities were excluded from the study [13, 25, 26]. Similarly, those with incomplete medical records were also excluded from the study. Using a prevalence of 37.5% (reported in previous study from the same study setting [27]), a target sample size of 358 was calculated using simple population formula.

### Microbiological investigations

The BVH follows standard microbiological techniques. The skin is disinfected using standard methods before collecting the venous blood samples. Afterwards, 1 to 3 ml blood sample is drawn from the neonate under aseptic condition and transferred to the BACTEC PedsPlus™ (Becton Dickinson, Ireland) culture vial. The vial is then incubated at 37 ^o^C for up to seven days. The culture vial is examined on daily basis for bacterial growth and turbidity [13]. The pure bacterial isolates are obtained using subculture technique and later the organisms are identified using methods described by Yadav et al. [28]. Antibiotic susceptibility testing is performed using disc diffusion method (Kirby-bauer method) as per Clinical and Laboratory Standards Institute (CLSI) guidelines [29].

### Outcomes variables

Treatment outcomes, antibiotic usage, and antibiotic resistance and sensitivity pattern were taken as the outcome variables.

Treatment outcomes were categorized into discharge (with treatment success), leave against medical advice (LAMA), discharge on request (DOR) and death.

- *Discharge*: Neonatal sepsis patient who was discharged from the ward after successfully completing the treatment.
- *Leave against medical advice*: Neonatal sepsis patient who left the ward without completing the treatment due to assorted reasons, such as financial issue or not satisfied with the treatment or hospital environment.
- *Discharge on request*: Neonatal sepsis patient who left the ward on request but without completing the treatment.
- *Death*: Patient died while on sepsis treatment.

### Data collection

A data collection form was developed after literature review [13, 30, 31]. The data collection form consisted of data on patient’s gender, residence, gestational age at birth, birth weight (very low birth weight, VLBW, < 1500 g; low birth weight, LBW, < 2500 g; normal birth weight, NBW, ≥ 2500 g), mode of child birth, diagnosis (EOS/LOS), treatment strategy, laboratory parameters, blood and urine culture, bacterial isolates, antimicrobial sensitivity and resistance pattern, and treatment outcomes [32]. The data were obtained from the medical records of the neonates and microbiology laboratory culture registers. Prior to the start of data collection, data collectors were guided and trained by the supervisor (MA) on how to collect data from the records.

### Data analysis

Statistical Package for Social Sciences (IBM Corp. released 2012.IBM SPSS statistics for windows, version 20.0, Armonk, NY: IBM Corp.) was used to analyze the data. The findings of the study were presented as counts (n) and proportions (%) for the categorical variables, and mean and standard deviation (SD) for the continuous variables. Chi- square test was used to test the statistically significant difference among the categorical variables. Due to high death rate among the study population, logistic regression analysis was used to identify the independent factors associated with it. The variables which were statistically significant in simple logistic regression analysis were entered into multivariable logistic regression analysis to identify the final predictors. A p-value of less than 0.05 was considered statistically significant.

### Ethical considerations

Pharmacy Research Ethics Committee (PREC) at the Islamia University of Bahawalpur (Reference: 74/S-2019-/PREC) approved the design and conduct of the study. At the study setting, the medical record of a patient is anonymized in a way that it only contains name of the patient and the hospital registration number without any further information. Therefore, the identity of the patients could not be disclosed to the data collectors or the principal investigator. Nevertheless, the PREC waived the requirement for informed consent.

## Results

During the study period, a total of 722 neonatal sepsis patients were enrolled at the study site. Out of these, 136 (18.83%) were excluded from the study because either their medical records were incomplete/missing or they were extremely low birth weight neonates. As a result, a total of 586 patients were included in the final analysis. Most of the patients were male (n = 398, 67.9%), while more than 75% of the patients were residents of rural areas (n = 454, 77.5%). Out of the total 586 patients, 328 (56%) were preterm, while 299 (51%) were born with low birth weight. A total of 415 (70.8%) neonates were diagnosed with EOS. C-section was relatively dominant mode of child delivery (n = 326, 55.6%). Only 52 (8.9%) neonates were culture positive, while a large number of population (n = 414, 70.6%) were culture negative (Table 1).

**Table 1:** Sociodemographic and clinical characteristics of the patients (N = 586).

Variable	n (%)
**Gender**	
Male	398 (67.9)
Female	188 (32.1)
**Gestational age at birth**	
Preterm (< 37 weeks of gestation)	328 (56)
Term	258 (44)
**Birth weight**	
Very low birth weight	131 (22.4)
Low birth weight	299 (51)
Normal birth weight	156 (26)
**Diagnosis**	
Early onset sepsis	415 (70.8)
Late onset sepsis	171 (29.2)
**Mode of child birth**	
Spontaneous vaginal delivery	260 (44.4)
Cesarean section	326 (55.6)
**Residence**	
Rural	454 (77.5)
Urban	132 (22.5)
**Length of hospital stay**	
≤6 days	262 (44.7)
≥6 days	324 (55.3)
**Culture status**	
Culture positive	52 (8.9)
Culture negative	414 (70.6)
Specimen not sent for culture	120 (20.5)
**Specimen type for culture**	
Blood	338 (57.7)
Urine	128 (21.8)

### Antibiotics initially started among the neonatal sepsis patients

At the start of treatment, most of the patients (n = 484, 82.6%) were treated with amikacin+cefotaxime, while 102 (17.4%) patients were given amikacin+ceftriaxone as a start treatment.

### Bacterial isolates identified among the neonatal sepsis patients

A total of 52 specimens were culture positive. Among them, *Klebsiella* species were dominant (n = 19, 36.5%) followed by *E*. *coli* (n = 15, 28.8%). *Staphylococcus aureus* was identified in eight (15.4%) specimens (Table 2).

**Table 2:** Type of bacteria identified in the culture positive patients.

Bacterial isolates	Specimen type		Total n (%)
Gram-negative	Blood n	Urine n	
*Klebsiella species*	15	4	19 (36.5)
*E*.*coli*	4	11	15 (28.8)
*Citrobacter species*	5	-	5 (9.6)
*Pseudomonas aeruginosa*	3	-	3 (5.8)
*Enterobacter species*	1	-	1 (1.9)
*Serratia species*	1	-	1 (1.9)
**Gram-positive**			
*Staphylococcus aureus*	7	1	8 (15.4)
**Total**			**52 (100)**

### Antibiotic sensitivity and resistance pattern against bacterial isolates

Out of the total 52 positive cultures, cefotaxime was tested in 35 isolates (34 gram-negative, one gram-positive). Out of these, 27 (77.1%) gram-negative and one (2.9%) gram positive isolates were resistant to this antibiotic. Similarly, ceftriaxone was tested in 39 isolates and it showed almost similar resistance pattern to cefotaxime. Amikacin was tested in 41 isolate (40 gram-negative and one gram-positive) and it was resistant in 15 (36.6%) gram negative and one (0.2%) gram positive isolates. Imipenem, piperacillin+tazobactam, vancomycin and linezolid showed better sensitivity against the bacterial isolated (Table 3). For details on number of resistant drugs against each of the identified bacteria, please refer to S1 File.

**Table 3:** Overall status of antibiotic resistance and sensitivity against bacterial isolates.

Antibiotic tested	Gram negative		Gram positive	
	R (%)	S (%)	R (%)	S (%)
Amoxicillin + Clavulanic acid (n = 41)	26 (63.4)	12 (29.3)	2 (4.9)	1 (2.4)
Piperacillin+ Tazobactam (n = 46)	1 (1.2)	38 (82.6)	0 (0)	7 (15.2)
Imipenem (n = 38)	0 (0)	37 (97.8)	0 (0)	1 (2.6)
Cefoperazone + sulbactam (n = 41)	7 (17)	34 (82.9)	0 (0)	0 (0)
Cefepime (n = 36)	21 (58)	15 (41.7)	0 (0)	0 (0)
Cefixime (n = 27)	24 (88.9)	3 (11.1)	0 (0)	0 (0)
Ceftazidime (n = 29)	18 (62)	10 (34.5)	0 (0)	1 (3.4)
Cefotaxime (n = 35)	27 (77.1)	7 (20)	0 (0)	1 (2.9)
Ceftriaxone (n = 39)	30 (76.9)	8 (20.5)	0 (0)	1 (2.5)
Ciprofloxacin (n = 40)	19 (47.5)	20 (50)	0 (0)	1 (2.5)
Levofloxacin (n = 41)	12 (30.8)	28 (71.8)	0 (0)	1 (2.5)
Amikacin (n = 41)	15 (36.6)	25 (61)	0 (0)	1 (0.2)
Vancomycin (n = 9)	0 (0)	2 (22.2)	0 (0)	7 (77.8)
Linezolid (n = 8)	0 (0)	1(12.5)	0 (0)	7 (87.5)
Clarithromycin (n = 8)	1 (1.2)	0 (0)	1(1.2)	6 (75)
Oxacillin (n = 4)	1 (25)	0 (0)	0 (0)	3 (75)
Metronidazole (n = 6)	1 (16.7)	0 (0)	4 (66.7)	1 (16.7)
Meropenem (n = 1)	0 (0)	0 (0)	0 (0)	1 (100)

Note: Calculations were based on number of samples with positive bacterial growth and in which specific antibiotic was tested for sensitivity and resistance against antibiotic

### Percentage resistance of bacterial isolates against antibiotics

Among the most common gram negative bacterial isolates (i.e., *Klebsiella species and E*. *coli)* identified in this study, highest level of resistance was seen against all tested cephalosporins, including those started as an empiric therapy (i.e., cefotaxime, ceftriaxone). 11 (66.1%) *Klebsiella* isolates were resistant to amikacin, but only one (7.7%; out of 12) *E*. *coli* isolates were resistant to amikacin. Most common gram negative isolates showed low level of resistance against piperacillin+tazobactam and imipenem (Table 4).

**Table 4:** Percentage resistance of bacterial isolates against antibiotics.

Antibiotic	*Klebsiella species*		*E*.*coli*		*Citrobacter species*		*Pseudomonas aerugenosa*		*Enterobacter species*		*Serratia species*		*Staphylococcus aureus*	
	R/(R+S)	R%	R/(R+S)	R%	R/(R+S)	R%	R/(R+S)	R%	R/(R+S)	R%	R/(R+S)	R%	R/(R+S)	R%
Amoxicillin + Clavulanic Acid	12/ (12+3)	80	10 / (10+5)	66.7	3 / (3+1)	75	0 / (0+2)	0	1 / (1+0)	100	0 / (0+1)	0	2 / (2+1)	66.7
Piperacillin+ Tazobactam	1 / (1+15)	6.25	0 / (0+13)	0	0 / (0+5)	0	0 / (0+3)	0	0 / (0+1)	0	0 / (0+1)	0	0 / (0+7)	0
Imipenem	0 / (0+15)	0	0 / (0+13)	0	0 / (0+4)	0	0 / (0+3)	0	0 / (0+1)	0	0 / (0+1)	0	0 / (0+1)	0
Cefoperazone + Sulbactam	5 / (5+13)	27.8	1 / (1+12)	7.7	1 / (1+4)	20	0 / (0+3)	0	0 / (0+1)	0	0 / (0+1)	0	0 / (0+0)	-
Cefepime	9 / (9+5)	64.3	11 / (11+3)	78.6	1 / (1+3)	25	0 / (0+2)	0	0 / (0+1)	0	0 / (0+1)	0	0 / (0+0)	-
Cefixime	9 / (9+0)	100	10 / (10+1)	90.9	2 / (2+0)	100	2 / (2+1)	66.7	1 / (1+0)	100	0 / (0+1)	0	0 / (0+0)	-
Ceftazidime	8 / (8+3)	72.7	9 / (9+2)	81.8	1 / (1+1)	50	0 / (0+2)	0	0 / (0+1)	0	0 / (0+1)	0	0 / (0+1)	0
Cefotaxime	15 / (15+2)	88.2	12/ (12+1)	92.3	0 / (0+3)	0	0 / (0+1)	0	0 / (0+0)	-	0 / (0+0)	-	0 / (0+1)	0
Ceftriaxone	16 / (16+2)	88.9	12 / (12+2)	85.7	2 / (2+3)	40	0 / (0+0)	-	0 / (0+0)	-	0 / (0+1)	0	0 / (0+1)	0
Ciprofloxacin	8 / (8+10)	44.4	10 / (10+3)	76.9	0 / (0+4)	0	1 / (1+2)	33.3	0 / (0+1)	0	0 / (0+0)	-	0 / (0+1)	0
Levofloxacin	5 / (5+14)	26.3	6 / (6+6)	50	0 / (0+4)	0	1 / (1+2)	33.3	0 / (0+1)	0	0 / (0+1)	0	0 / (0+1)	0
Amikacin	11 / (11+7)	66.1	1 / (1+12)	7.7	2 / (2+2)	50	1 / (1+2)	33.3	0 / (0+1)	0	0 / (0+1)	0	0 / (0+1)	0
Vancomycin	0 / (0+0)	-	0 / (0+2)	-	0 / (0+0)	-	0 / (0+0)	-	0 / (0+0)	-	0 / (0+0)	-	0 / (0+7)	0
Linezolid	0 / (0+0)	-	0 / (0+1)	-	0 / (0+0)	-	0 / (0+0)	-	0 / (0+0)	-	0 / (0+0)	-	0 / (0+7)	0
Clarithromycin	1 / (1+0)	100	0 / (0+0)	-	0 / (0+0)	-	0 / (0+0)	-	0 / (0+0)	-	0 / (0+0)	-	1 / (1+6)	14.3
Oxacillin	0 / (0+0)	-	1 / (1+0)	100	0 / (0+0)	-	0 / (0+0)	-	0 / (0+0)	-	0 / (0+0)	-	0 / (0+3)	0
Metronidazole	0 / (0+0)	-	1 / (1+0)	100	0 / (0+0)	-	0 / (0+0)	-	0 / (0+0)	-	0 / (0+0)	-	4 / (4+1)	80
Meropenem	0 / (0+0)	-	0 / (0+0)	-	0 / (0+0)	-	0 / (0+0)	-	0 / (0+0)	-	0 / (0+0)	-	0 / (0+1)	0

Out of the 52 specimen which showed bacterial growth, Klebsiella = 19, E.coli = 15, pseudomonas = 3, Enterobacter = 5, Citrobacter = 5, Serratia = 1, Staphylococcus aureus = 8; R = number of resistant isolates; R% = percentage of resistant isolates; S = number of sensitive isolate

### Modification in antibiotic treatment among the neonatal sepsis patients

Among the total 586 patients, antibiotic treatment was modified in 175 (29.9%) patients either based on physician’s judgment and/or culture and sensitivity results. Out of 175 patients in whom therapy was modified, amikacin+cefotaxime was prescribed as a start treatment in 168 patients (96%), while remaining seven (4%) patients were on amikacin+ceftriaxone. With regard to modifications, amikacin+ceftriaxone was started as a modified treatment in 71 (40.6%) cases, while cefoperazone+sulbactam+ampicillin and vancomycin+ceftriaxone combinations were started as modified treatment in 29 (16.6%) cases each. Interestingly, only 27 (15.4%) treatment modifications were based on culture and sensitivity results, while the remaining 148 (84.6%) modifications were made on physician’s judgment (Table 5).

**Table 5:** Modification in the antibiotic treatment based on physician’s judgment, and culture and sensitivity results.

Modified treatment	Modified after c/s n (%)	Modified after negative culture n (%)	c/s not done but treatment modified n (%)	Total n (%)
Amikacin+ceftriaxone	3 (4.2)	60 (84.5)	8 (11.3)	71 (40.6)
Vancomycin+ceftriaxone	6 (20.7)	22 (75.9)	1 (3.4)	29 (16.6)
Vancomycin+ meropenem	6 (66.7)	3 (33.3)	0 (0)	9 (5.1)
Cefoperazone+sulbactam+Ampicillin	0 (0)	25 (86.2)	4 (13.8)	29 (16.6)
Cefoperazone+sulbactam+B.penicillin	0 (0)	8 (100)	0 (0)	8 (4.6)
Imipenem+amikacin	5 (100)	0 (0)	0 (0)	5 (2.8)
Imipenem+cefotaxime	2 (100)	0 (0)	0 (0)	5 (1.1)
Imipenem+ceftriaxone	5 (100)	0 (0)	0 (0)	2 (2.8)
Cefoperazone+sulbactam+Amikacin	0 (0)	15 (88.2)	2 (11.8)	17 (9.7)
**Total**	**27 (15.4)**	**133 (76)**	**15 (8.6)**	**175 (100)**

### Treatment outcomes among the neonatal sepsis patients

With regard to treatment outcomes among the total 586 patients, 280 (47.8%) were discharged (with treatment success), 122 (20.8%) LAMA, 61 (10.4%) were DOR and 123 (21%) died during treatment (Table 6). A detailed description of treatment outcomes with regard to patients’ characteristics is provided in S2 File.

**Table 6:** Treatment outcomes among the patients.

Outcome	n (%)
Discharged (with treatment success)	280 (47.8)
Leave against medical advice	122 (20.8)
Discharge on request	61 (10.4)
Death	123 (21)

### Factors associated with death among neonatal sepsis patients

In simple logistic regression analysis, the factors which were significantly associated with neonatal death included; preterm delivery (OR 16.87; 95% CI 8.05, 35.35); sub-optimal birth weight (OR 7.86; 95% CI 3.58, 17.28), EOS (OR 6.81; 95% CI 3.37, 13.80), cesarean delivery (OR 2.06; 95% CI 1.35, 3.14) and length of hospital stay (OR 0.79; 95% CI 0.70, 0.89). In multivariable binary logistic regression, the factors which still remained significantly associated with neonatal death included; preterm delivery (AOR 9.59; 95% CI 4.41, 20.84); sub-optimal birth weight (AOR 5.13; 95% CI 2.19, 12.04), EOS (AOR 2.99; 95% CI 1.39, 6.41) and length of hospital stay (AOR 0.76; 95% CI 0.67, 0.88) (Table 7).

**Table 7:** Factors associated with death among neonatal sepsis patients: Multivariable binary logistic regression analysis.

Independent variable	B	S.E	p-value	AOR (95% CI)
Preterm delivery	2.26	.396	<0.0005	9.59 (4.41, 20.84)
Sub-optimal birth weight	1.63	.435	<0.0005	5.13 (2.19, 12.04)
Early onset sepsis	1.09	.389	.005	2.99 (1.39, 6.41)
Cesarean delivery	0.10	.252	.688	1.10 (0.68, 1.81)
Length of hospital stay*	-0.271	.070	<0.0005	0.76 (0.67, 0.88)

Note: Sub-optimal birth weight includes both very low birth weight and low birth weight

*entered as continuous variable; Hosmer and Lemeshow test (8.300), p = 0.405; Nagelkerke R Square (0.357); Model summary = Chi square (152.79), df (5), p <0.0005

**Fig 1:**
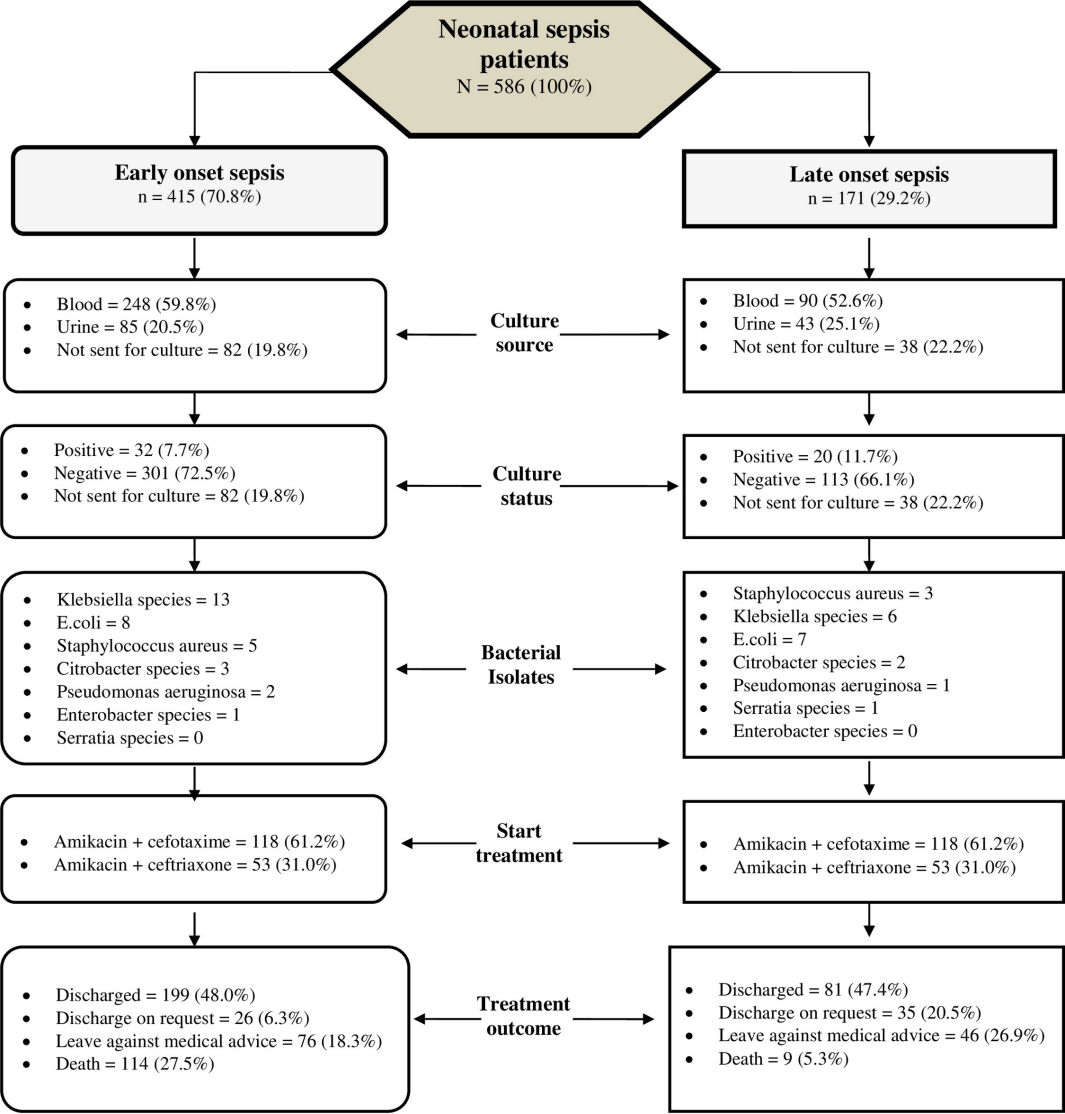
Differences in important study parameters between neonates with early and late onset sepsis.

Fig 1 presents the differences in the important study parameters with regard to diagnosis (i.e., EOS and LOS).

## Discussion

The lack of recognition of neonatal sepsis as a major public health concern is primarily attributable to paucity of detailed data, especially in developing nations [33]. To our knowledge, this is the first study that simultaneously assessed treatment outcomes, antibiotic use and its resistance pattern among neonatal sepsis patients in a tertiary care hospital of Pakistan. In most of the patients, diagnosis of sepsis was made on clinical findings rather than microbiological. We found that just over 47% (n = 280) of the patients were discharged from hospital after clinical improvements (i.e., discharged with treatment success). However, more than 30% (n = 183) of the patients left the pediatric wards either against the medical advice or on request. More than 20% (n = 123) of the neonatal sepsis patients died during the treatment which is alarming. *Klebsiella* species and *E*. *Coli* were the most commonly identified gram negative bacterial isolates and these showed high level of resistance against parenteral antibiotics started at the time of treatment. We also noted that most of the modifications in the treatment were based on physician’s judgement, but not on microbiological reports. This study also identified the independent factors associated with death among the neonatal sepsis patients.

In the current study, most of the neonatal patients (67.9%) were male and this is in agreement with the previous study conducted in Pakistan [34]. Greater susceptibility of male to suffer from infections might be due presence of single x (x) chromosome. The x chromosome has several genes that regulate the production of immunoglobulins. Females have double x (xx) chromosomes which lead to more production of immunoglobulins making them less vulnerable to infections [35]. Our study findings also showed that a greater percentage (77.5%) of the patients were from rural areas. Similarly, most of the neonatal sepsis patients were those who had less than optimal (VLBW and LBW) birth weight, and were suffering from EOS. These attributes are related to patient’s mother in a way that mothers living in rural areas of Pakistan might have poor socioeconomic status [20], unhealthy living environment [36], lack of healthcare facilities leading to unhygienic obstetric practices (intrapartum transmission from mothers) and poor nursery practices thus predisposing the newborns at the risk of infections [37]. Earlier studies have shown that preterm babies and those with less than optimal birth weight have underdeveloped immune system, which makes newborns susceptible to infections [13, 38, 39].

The results of our study revealed that diagnosis of the presumptive sepsis patients were made on the basis of physician’s judgment and laboratory investigations (CBC and CRP). For 120 patients, the physicians did not send the sample for bacteriological profile, and for the remaining 466 patients in whom bacteriology was requested, only 52 (11.7%; out of 446) were culture positive. Studies from other developing countries also reported low culture positivity rates among neonatal sepsis patients, for example, Bangladesh (9%), India (36%) and Egypt (43%) [12, 40, 41]. Low sensitivity of cultures might be due to wrong sampling collection and processing procedures, improper sample transportation, use of antibiotics prior to sample collection, slow growing bacteria, and viral, fungal or parasitic infections [12].

*Kelbsiella species* and *E*. *coli* were the most common gram negative and *Staphylococcus aureus* was the only gram positive bacterial isolate identified in this study. This finding is comparable to what was observed in a previous Pakistani study [11]. Studies from other developing countries like Nepal, Egypt, Ghana reported that the most common bacterial isolates among neonatal sepsis patients were *Klebsiella species*, *Pseudomona aeruginosa*, *E*. *coli*, *Enterobacter species*, *CoNS* and *Staphylococcus aureus* [12–14, 28]. In order to treat sepsis, the physicians in our study prescribed amikacin+cefotaxime combination in most of the patients (82.6%) followed by amikacin+cerftrixone combination (17.4%). Alarmingly, in this study, most of the commonly identified bacterial isolates showed high level of resistance against cephalosporins. Similarly, over 60% of *Klebsiella* isolates showed resistance against amikacin. A systematic review showed that *Klebsiella* species were highly resistant to cephalosporins (84%) in Asian countries [42]. Two other systematic reviews and meta-analysis also concluded that most common bacterial isolates responsible for causing neonatal sepsis in LMICs were resistant to or had reduced susceptibility to the WHO recommended ampicillin and gentamicin combination therapy and to third generation cephalosporins [15, 43].

It is important to note that despite being aware of the most common bacterial isolates and their resistance against the standard antibiotic therapy at the BVH, the physicians in the current study neither tailored the therapy according to need of the patient nor according to the WHO recommendations. In the current scenario, the WHO recommended ampicillin+gentamicin (first-line) and third generation cephalosporins (second-line) may not be the viable options because the most common bacterial isolates in the present study were resistant to penicillins and cephalosporins (Tables 3 and 4). However, piperacillin+tazobactam, imipenem, vancomycin and linezolid showed better sensitivity against these bacterial isolates. Studies from other developing countries like India, Nepal, Egypt showed that these aforementioned antibiotics showed promising results in their healthcare settings in terms of antibacterial activity against most common bacterial isolates involved in neonatal sepsis [44–47]. Selection of empiric antibacterial therapy according to antibiotic resistance pattern in locally prevalent bacterial isolates is consistent with the recommendations of most of the neonatal sepsis treatment guidelines [15]. A study from Israel provided the evidence that the piperacillin+tazobactam combination plus amikacin were safe to use in neonatal sepsis patients and effectively/successfully eradicated (>90%) gram-negative and gram-positive organisms [48]. Similarly, a study from the US also advocated the superiority of piperacillin+tazobactam over ampicillin+gentamicin in terms of effectiveness and safety profile among neonatal sepsis patients [49]. Many guidelines, for example, Surviving Sepsis Campaign, British National Formulary (children), BMJ Clinical Evidence and American Academy of Pediatrics) have suggested the judicious use (benefits outweigh harmful effects) of piperacillin+tazobactam, imipenem, vancomycin and linezolid [15]. Based on antibacterial spectrum of these antibiotics, piperacillin+tazobactam or imipenem seems to be the better options for gram-negative and gram-positive bacteria [50] and vancomycin or linezolid for gram-positive bacteria [51]. However, considering the probability of adverse consequences arising from the long-term use of broad spectrum antibiotic [13], mandatory culture and susceptibility investigations are crucial in-order to timely switch to narrow-spectrum antibiotics.

With regard to treatment outcomes, nearly 50% of our patients were discharged from the wards after successful completion of treatment. In contrast, an Ethiopian study showed that more than 80% of the patients were discharged after successfully completing the treatment [30]. Importantly, 23% of neonatal sepsis patients left the BVH against the medical advice. Previous studies form Pakistan and India also stated that 25% of neonatal patients left the pediatric wards against the medical advice [52, 53]. Some of the reasons which may provoke the patients to LAMA include, financial problems, not satisfied with the medical care, preferences for other hospitals and communication gap between the health care providers and parents. Alarmingly, 21% of the neonatal sepsis patients enrolled in this study died during the treatment, which demand better treatment compliance through regular monitoring of CRP levels, review of culture and frequent consideration of other physiological parameters to timely start interventions [54, 55]. Nevertheless, the mortality rated reported in our study is almost similar to the mortality rate reported in Egypt (22%) and Iran (19%) [12, 56]. Similarly, data retrieved from multiple studies conducted in various developing countries presented a wide range of neonatal mortality rate related to infection (8–80%) [57]. In developing nations, including our setting [27, 58, 59], high neonatal mortality rates are widely known to be attributed to poor quality management of seriously ill neonates due to absence of neonatal specialists and neonatal intensive care units, delays in newborn resuscitation, unavailability of warming devices, and non-utilization of antenatal care.

Careful selection of antibiotics in neonatal sepsis patients can greatly reduce the risk of death [60]. For example, Table 5 shows that the physicians modified antibiotic treatment in 175 (29.9%) patients either based on their clinical judgement and/or culture and sensitivity results. Further analysis of the data revealed that none of the patients died in whom imipenem (in combination) was started as a modified treatment. Moreover, one patient died among those in whom vancomycin (in combination) was started as a modified treatment. Similarly, cefoperazone+sulbactam (in combination with other antibiotics) also showed promising results (data not shown in results, for details please refer to S3 and S4 Files). These findings clearly signify the importance of microbiological data in tailoring the antibiotic regimen for neonatal sepsis patients and ultimately curbing sepsis related mortality at our study setting. Beside the appropriate selection of antibiotics, there are some other factors which may predict mortality among neonatal sepsis patients. For example, in our study, preterm neonates (9.59 times), those having suboptimal birth weight (5.13 times) and suffering from EOS (2.99 times) had higher chances of dying compared to term neonates, those having optimal birth weight and had LOS, respectively. As discussed earlier, neonates with these particularities have weakened immune system thus making them more prone to death due to sepsis [4, 61]. This study also demonstrated that neonatal sepsis patients with longer hospital stay had lesser chances of death than those with shorter hospital stay. One possible reason for this finding could be that the severely ill patients died earlier than those who had mild to moderate infection.

Our study has a few limitations. First, this study was conducted at only one tertiary care hospital of the Punjab province of Pakistan. Therefore, the findings should be generalized with care for whole of Pakistan as type of pathogens and antibiotic use pattern may vary across the country. Second, due to retrospective nature of the study, we were unable to capture some of the important variables (not available in patient files) (for example, maternal variables, prior use of antibiotics by the neonates, etc.) which could have affected the treatment outcomes. Third, small sample and low culture positivity rates are few other limitations of the study. Hence, multicenter studies with larger sample size are required to validate our findings. Moreover, future studies should also report treatment outcomes, and antibiotic use and susceptibility pattern among neonatal sepsis patients with meningitis, pneumonia etc.

## Conclusion

Just under half of the total neonatal sepsis patients successfully completed the treatment (i.e., discharged with treatment success), whereby the mortality rate was high in the study cohort. *Klebsiella species*, *E*. *coli* and *Staphylococcus aureus* were the most common bacterial isolates identified in the study. Most of the bacterial isolates showed high level of resistance against empiric treatment. Preterm, less than optimal birth weight and EOS babies were more likely to die from sepsis. Given the antibacterial spectrum of the tested antibiotics, the combination of piperacillin+tazobactam with amikacin could be recommended as an empiric therapy for neonatal sepsis patients at our study setting. Imipenem in combination with other antibiotics may also be considered as a treatment option.

## Supporting information

S1 File Number of resistant drugs with regard to identified bacteria. (DOCX)

S2 File Treatment outcomes with regard to the patient characteristics. (DOCX)

S3 File Percentage of death with regard to modified treatment. (DOCX)

S4 File Number of deaths with regard to culture status. (DOCX)

S1 Data (SAV)

## References

[1] Chaurasia S, Sivanandan S, Agarwal R, Ellis S, Sharland M, Sankar MJ. Neonatal sepsis in South Asia: huge burden and spiralling antimicrobial resistance. BMJ. 2019;364:k5314 10.1136/bmj.k5314 30670451 PMC6340339

[2] Shane AL, Sanchez PJ, Stoll BJ. Neonatal sepsis. Lancet. 2017;390(10104):1770–80. 10.1016/S0140-6736(17)31002-4 28434651

[3] Singh M, Gray CP. Neonatal sepsis. StatPearls [Internet]: StatPearls Publishing; 2019. 30285373

[4] Simonsen KA, Anderson-Berry AL, Delair SF, Davies HD. Early-onset neonatal sepsis. Clinical microbiology reviews. 2014;27(1):21–47. 10.1128/CMR.00031-13 24396135 PMC3910904

[5] Fleischmann-Struzek C, Goldfarb DM, Schlattmann P, Schlapbach LJ, Reinhart K, Kissoon N. The global burden of paediatric and neonatal sepsis: a systematic review. The Lancet Respiratory Medicine. 2018;6(3):223–30. 10.1016/S2213-2600(18)30063-8 29508706

[6] World Bank. Mortality rate, neonatal (per 1,000 live births)—Pakistan 2020 [Available from: https://data.worldbank.org/indicator/SH.DYN.NMRT?locations=PK.

[7] Ministry of Finance. Economic Survey 2018–19. 2019.

[8] United Nations International Children's Emergency Fund. Neonatal mortality 2020 [Available from: https://data.unicef.org/topic/child-survival/neonatal-mortality/.

[9] Lawn JE, Blencowe H, Oza S, You D, Lee AC, Waiswa P, et al Every Newborn: progress, priorities, and potential beyond survival. The Lancet. 2014;384(9938):189–205. 10.1016/S0140-6736(14)60496-7 24853593

[10] United Nations International Children's Emergency Fund. Maternal and newborn health disparities: Pakistan 2015 [Available from: data.unicef.org › wp-content › uploads › country_profiles › Pakistan.

[11] Khaliq A, Rahman SU, Gul S, Khan MA, Shaheryar ZA, Zaman M, et al Emerging antimicrobial resistance causing therapeutic failure in neonatal sepsis. Biocatalysis and Agricultural Biotechnology. 2019;20:101233.

[12] Mohsen L, Ramy N, Saied D, Akmal D, Salama N, Haleim MMA, et al Emerging antimicrobial resistance in early and late-onset neonatal sepsis. Antimicrobial Resistance & Infection Control. 2017;6(1):63 10.1186/s13756-017-0225-9 28630687 PMC5470277

[13] Pokhrel B, Koirala T, Shah G, Joshi S, Baral P. Bacteriological profile and antibiotic susceptibility of neonatal sepsis in neonatal intensive care unit of a tertiary hospital in Nepal. BMC pediatrics. 2018;18(1):208 10.1186/s12887-018-1176-x 29950162 PMC6020420

[14] Aku FY, Akweongo P, Nyarko K, Sackey S, Wurapa F, Afari EA, et al Bacteriological profile and antibiotic susceptibility pattern of common isolates of neonatal sepsis, Ho Municipality, Ghana-2016. Maternal health, neonatology and perinatology. 2018;4(1):2 10.1186/s40748-017-0071-z 29403648 PMC5778684

[15] Fuchs A, Bielicki J, Mathur S, Sharland M, Van Den Anker JN. Reviewing the WHO guidelines for antibiotic use for sepsis in neonates and children. Paediatrics and international child health. 2018;38(sup1):S3–S15. 10.1080/20469047.2017.1408738 29790842 PMC6176768

[16] Malik I, Atif M. Global menace of superbugs: Time to consider a "Pharmacist led One Health Approach" to counteract the crisis. Res Social Adm Pharm. 2020;16(6):848–9. 10.1016/j.sapharm.2020.02.018 32201107

[17] Laxminarayan R, Matsoso P, Pant S, Brower C, Røttingen J-A, Klugman K, et al Access to effective antimicrobials: a worldwide challenge. The Lancet. 2016;387(10014):168–75. 10.1016/S0140-6736(15)00474-2 26603918

[18] Malik I, Atif M, Riaz F, Asghar S, Ahmad N. Pediatric Antibiotic Pack Size Compliance With the Dosage Regimen: A Descriptive Study. Therapeutic Innovation & Regulatory Science. 2020;54(3):492–506. 10.1007/s43441-019-00081-7 33301133

[19] Atif M, Asghar S, Mushtaq I, Malik I, Amin A, Babar Z-U-D, et al What drives inappropriate use of antibiotics? A mixed methods study from Bahawalpur, Pakistan. Infect Drug Resist. 2019;12:687–99. 10.2147/IDR.S189114 30988635 PMC6440533

[20] Atif M, Malik I, Asif M, Qamar-Uz-Zaman M, Ahmad N, Scahill S. Drug safety in Pakistan In: Al-Worafi Y, editor. Drug Safety in Developing Countries: Achievements and Challenges. India: Elsevier; 2020 p. 287–316.

[21] Hussain M, Aurakzai AA, Irshad M, Ullah I. Neonatal Sepsis; Frequency of Various Bacteria and their Antibiotic Sensitivity in Neonatal Sepsis. Professional Medical Journal. 2018;25(11).

[22] Nizami N, Quddusi AI, Razzaq A, Amjad A, Nazir S. Neonatal Sepsis: An Evaluation of Bacteriological Spectrum and Antibiotic Susceptibilities in NICU of Children Hospital Multan. Clinical Characteristics and Risk Factors of Candidemia in Tertiary Care Hospital. 2015;6:855.

[23] Atif M, Sarwar MR, Azeem M, Umer D, Rauf A, Rasool A, et al Assessment of WHO/INRUD core drug use indicators in two tertiary care hospitals of Bahawalpur, Punjab, Pakistan. Journal of pharmaceutical policy and practice. 2016;9(1):27 10.1186/s40545-016-0076-4 27688887 PMC5034517

[24] Kanodia P, Yadav SK, Bhatta NK, Singh RR. Disease profile and outcome of newborn admitted to neonatology unit of BPKIHS. Journal of College of Medical Sciences-Nepal. 2015;11(3):20–4.

[25] Afrin M, Siddique MA, Ahmed AA, Islam MN, Sarker PC, Showkath MS, et al Neonatal Septicemia: Isolation, Identification and Antibiotic Sensitivity Pattern of Bacteria in a Tertiary Hospital in Bangladesh. Faridpur Medical College Journal. 2016;11(2):58–61.

[26] Mhada TV, Fredrick F, Matee MI, Massawe A. Neonatal sepsis at Muhimbili National Hospital, Dar es Salaam, Tanzania; aetiology, antimicrobial sensitivity pattern and clinical outcome. BMC public health. 2012;12(1):904. 23095365 10.1186/1471-2458-12-904 PMC3503784

[27] Mazhar A, Rehman A, Sheikh MA, Naeem MM, Qaisar I, Mazhar M. Neonates—a neglected paediatric age group. JPMA. 2011;61(625). 22204232

[28] Yadav NS, Sharma S, Chaudhary DK, Panthi P, Pokhrel P, Shrestha A, et al Bacteriological profile of neonatal sepsis and antibiotic susceptibility pattern of isolates admitted at Kanti Children’s Hospital, Kathmandu, Nepal. BMC research notes. 2018;11(1):301 10.1186/s13104-018-3394-6 29764503 PMC5952417

[29] Clinical and Laboratory Standards Institute. Performance standards for antimicrobial susceptibility testing (M100-S23) Wayne, PA 19087 USA2013 [Available from: https://www.facm.ucl.ac.be/intranet/CLSI/CLSI-M100S23-susceptibility-testing-2013-no-protection.pdf. 10.1128/JCM.00213-21 PMC8601225 34550809

[30] Tewabe T, Mohammed S, Tilahun Y, Melaku B, Fenta M, Dagnaw T, et al Clinical outcome and risk factors of neonatal sepsis among neonates in Felege Hiwot referral Hospital, Bahir Dar, Amhara Regional State, North West Ethiopia 2016: a retrospective chart review. BMC research notes. 2017;10(1):265 10.1186/s13104-017-2573-1 28693597 PMC5504561

[31] Fleming PF, Forster D, Savage T, Sudholz H, Jacobs SE, Daley AJ. Evaluating suspected sepsis in term neonates. Journal of Neonatal Nursing. 2012;18(3):98–104.

[32] Getabelew A, Aman M, Fantaye E, Yeheyis T. Prevalence of neonatal sepsis and associated factors among neonates in neonatal intensive care unit at selected governmental hospitals in Shashemene Town, Oromia Regional State, Ethiopia, 2017. International journal of pediatrics. 2018;2018. 10.1155/2018/7801272 PMC6098917 30174698

[33] Agarwal R, Sankar J. Characterisation and antimicrobial resistance of sepsis pathogens in neonates born in tertiary care centres in Delhi, India: a cohort study. The Lancet Global Health. 2016;4(10):e752–e60. 10.1016/S2214-109X(16)30148-6 27633433

[34] Saleem AF, Qamar FN, Shahzad H, Qadir M, Zaidi AK. Trends in antibiotic susceptibility and incidence of late-onset Klebsiella pneumoniae neonatal sepsis over a six-year period in a neonatal intensive care unit in Karachi, Pakistan. International Journal of Infectious Diseases. 2013;17(11):e961–e5. 10.1016/j.ijid.2013.04.007 23759260

[35] Pius S, Bello M. Neonatal Septicaemia in Poor Resource Settings. Pediatric Infect Dis. 2017;2(34):2573–0282.100034.

[36] Atif M, Malik I. Why is Pakistan vulnerable to COVID-19 associated morbidity and mortality? A scoping review. Int J Health Plann Manag. 2020 10.1002/hpm.3016 32700410 PMC7404956

[37] Noh J-W, Kim Y-m, Akram N, Yoo K-B, Cheon J, Lee LJ, et al Impact of socio-economic factors and health information sources on place of birth in Sindh Province, Pakistan: A secondary analysis of cross-sectional survey data. International journal of environmental research and public health. 2019;16(6):932 10.3390/ijerph16060932 30875876 PMC6466183

[38] Zaidi AK, Huskins WC, Thaver D, Bhutta ZA, Abbas Z, Goldmann DA. Hospital-acquired neonatal infections in developing countries. The Lancet. 2005;365(9465):1175–88. 10.1016/S0140-6736(05)71881-X 15794973

[39] Hafsa A, Fakruddin M, Hakim M, Sharma J. Neonatal bacteremia in a neonatal intensive care unit: analysis of causative organisms and antimicrobial susceptibility. Bangladesh Journal of Medical Science. 2011;10(3):187–94.

[40] Raha BK, Baki MA, Begum T, Nahar N, Jahan N, Begum M. Clinical, bacteriological profile & outcome of neonatal sepsis in a tertiary care hospital. Medicine Today. 2014;26(1):18–21.

[41] Sarasam S, Geetha S, Kumar S. Clinical and epidemiological profile of neonatal sepsis in referral care NICU in South Kerala. Journal of Medical Science And clinical Research. 2017;4(3):19327–33.

[42] Le Doare K, Bielicki J, Heath PT, Sharland M. Systematic review of antibiotic resistance rates among gram-negative bacteria in children with sepsis in resource-limited countries. Journal of the pediatric infectious diseases society. 2014;4(1):11–20. 10.1093/jpids/piu014 26407352

[43] Downie L, Armiento R, Subhi R, Kelly J, Clifford V, Duke T. Community-acquired neonatal and infant sepsis in developing countries: efficacy of WHO's currently recommended antibiotics—systematic review and meta-analysis. Archives of disease in childhood. 2013;98(2):146–54. 10.1136/archdischild-2012-302033 23142784

[44] Shrestha S, Adhikari N, Rai B, Shreepaili A. Antibiotic resistance pattern of bacterial isolates in neonatal care unit. Journal of the Nepal medical Association. 2010;50(180). 22049890

[45] El Haleim MA, Nawar NN, El Rahman EA, Hussein HA, Kamel NM. Epidemiologic and micro-bacteriologic study of neonatal septicaemia in Cairo University neonatal intensive care units. Res J Med Med Sci. 2009;4(1):67–77.

[46] Pooja R, Sowmya K, Shrikala B, Radhakrishna M, Keerthiraj B. A spectrum of bacterial pathogens and its antibiotic susceptibility pattern isolated from neonatal sepsis in an NICU in a Government Pediatric Hospital. Int Res J Biological Sci. 2015;4:50–4.

[47] Thapa S, Sapkota LB. Changing Trend of Neonatal Septicemia and Antibiotic Susceptibility Pattern of Isolates in Nepal. International journal of pediatrics. 2019;2019 10.1155/2019/3784529 30881464 PMC6381565

[48] Flidel-Rimon O, Friedman S, Leibovitz E, Shinwell ES. The use of piperacillin/tazobactam (in association with amikacin) in neonatal sepsis: efficacy and safety data. Scandinavian journal of infectious diseases. 2006;38(1):36–42. 10.1080/00365540500372879 16338836

[49] Chong E, Reynolds J, Shaw J, Forur L, Delmore P, Uner H, et al Results of a two-center, before and after study of piperacillin–tazobactam versus ampicillin and gentamicin as empiric therapy for suspected sepsis at birth in neonates < 1500 g. Journal of Perinatology. 2013;33(7):529–32. 10.1038/jp.2012.169 23328923

[50] Joly-Guillou M-L, Kempf M, Cavallo J-D, Chomarat M, Dubreuil L, Maugein J, et al Comparative in vitro activity of Meropenem, Imipenem and Piperacillin/tazobactam against 1071 clinical isolates using 2 different methods: a French multicentre study. BMC infectious diseases. 2010;10(1):72 10.1186/1471-2334-10-72 20298555 PMC2845586

[51] Hashemian SMR, Farhadi T, Ganjparvar M. Linezolid: a review of its properties, function, and use in critical care. Drug design, development and therapy. 2018;12:1759. 10.2147/DDDT.S164515 PMC6014438 29950810

[52] Yasmeen S, Waheed KAI, Gul R. Spectrum of neonatal admissions and their outcome in a tertiary Care hospital. Pakistan Armed Forces Medical Journal. 2017;67(6):1044–49.

[53] Devpura B, Bhadesia P, Nimbalkar S, Desai S, Phatak A. Discharge against medical advice at neonatal intensive care unit in Gujarat, India. International journal of pediatrics. 2016;2016 10.1155/2016/1897039 28003834 PMC5143712

[54] MacCarrick T, Magee A, Jones S, Gravell E, Davies P, Abelian A. G134(P) Improving compliance with national guidelines for early onset neonatal sepsis. 2018;103(Suppl 1):A55-A.

[55] Ekman B, Paudel P, Basnet O, Kc A, Wrammert J. Adherence to World Health Organisation guidelines for treatment of early onset neonatal sepsis in low-income settings; a cohort study in Nepal. BMC Infectious Diseases. 2020;20(1):666 10.1186/s12879-020-05361-4 32912140 PMC7487985

[56] Movahedian A, Moniri R, Mosayebi Z. Bacterial culture of neonatal sepsis. Iranian Journal of Public Health. 2006:84–9.

[57] Thaver D, Zaidi AK. Burden of neonatal infections in developing countries: a review of evidence from community-based studies. The Pediatric infectious disease journal. 2009;28(1):S3–S9. 10.1097/INF.0b013e3181958755 19106760

[58] Bhutta ZA, Memon ZA, Soofi S, Salat MS, Cousens S, Martines J. Implementing community-based perinatal care: results from a pilot study in rural Pakistan. Bulletin of the World Health Organization. 2008;86(6):452–9. 10.2471/blt.07.045849 18568274 PMC2647462

[59] Wall SN, Lee AC, Niermeyer S, English M, Keenan WJ, Carlo W, et al Neonatal resuscitation in low-resource settings: what, who, and how to overcome challenges to scale up? International journal of gynaecology and obstetrics: the official organ of the International Federation of Gynaecology and Obstetrics. 2009;107 Suppl 1(Suppl 1):S47–62, s3-4. 10.1016/j.ijgo.2009.07.013 PMC2875104 19815203

[60] Korang SK, Safi S, Gluud C, Lausten-Thomsen U, Jakobsen JC. Antibiotic regimens for neonatal sepsis-a protocol for a systematic review with meta-analysis. Systematic Reviews. 2019;8(1):1–13. 10.1186/s13643-018-0916-1 31805993 PMC6896287

[61] Hornik CP, Fort P, Clark RH, Watt K, Benjamin DK Jr, Smith PB, et al Early and late onset sepsis in very-low-birth-weight infants from a large group of neonatal intensive care units. Early human development. 2012;88:S69–S74. 10.1016/S0378-3782(12)70019-1 22633519 PMC3513766

