# Supplementary material for: Treatment outcomes, antibiotic use and its resistance pattern among neonatal sepsis patients attending Bahawal Victoria Hospital, Pakistan
Source: PLoS One. 2021 Jan 13;16(1):e0244866. doi: 10.1371/journal.pone.0244866 (PMC7806133; doi:10.1371/journal.pone.0244866)
Supplement: S1 File — (DOCX) [file pone.0244866.s001.docx]

**S1 File: Number of resistant antibiotics with regard to identified bacteria**

| **Bacterial isolates** | **Number of antibiotics** | |
| --- | --- | --- |
|  | **Minimum** | **Maximum** |
| **Gram-negative** |  |  |
| *Klebsiella species* | 1 | 10 |
| *E.coli* | 1 | 8 |
| *Citrobacter species* | 1 | 5 |
| *Pseudomonas aeruginosa* | 1 | 3 |
| *Enterobacter species* | 2 | - |
| *Serratia species* | 1 | - |
| **Gram-positive** |  |  |
| *Staphylococcus aureus* | 1 | 2 |
