# Supplementary material for: Treatment outcomes, antibiotic use and its resistance pattern among neonatal sepsis patients attending Bahawal Victoria Hospital, Pakistan
Source: PLoS One. 2021 Jan 13;16(1):e0244866. doi: 10.1371/journal.pone.0244866 (PMC7806133; doi:10.1371/journal.pone.0244866)
Supplement: S2 File — (DOCX) [file pone.0244866.s002.docx]

**S2 File: Treatment outcomes with regard to the patient characteristics**

A statistically significant difference (i.e., p< 0.05) was found among term and preterm neonates with regard to discharge and death. Similarly, a statistically significant difference (i.e., p<0.005) was found among VLBW, LBW and NBW neonates with regard to all treatment outcome categories. Supplementary File 2 provides a full description of treatment outcomes with regard to patient characteristics and results of Chi-square test.

**S2 File: Treatment outcomes with regard to characteristics of the patients**

| **Characteristics** | **Outcomes** | | | | | | | |  |
| --- | --- | --- | --- | --- | --- | --- | --- | --- | --- |
|  | **Discharge**  **n=280** | **P-value** | **LAMA**  **n=122** | **P- value** | **DOR**  **n=61** | **P- value** | **Death**  **n=123** | **P- value** | **Total**  **n=586** |
| **Gender** |  | .340 |  | .448 |  | .128 |  | .506 |  |
| Male | 193 (48.5) |  | 84 (21.1) |  | 37 (9.3) |  | 84 (21.1) |  | 398(100) |
| Female | 87 (46.3) |  | 38 (20) |  | 24 (12.8) |  | 39 (20.7) |  | 188(100 |
| **Residence** |  | .249 |  | .236 |  | .184 |  | .220 |  |
| Rural | 213 (46.9) |  | 98 (21.6) |  | 44 (9.7) |  | 99 (21.8) |  | 454(100) |
| Urban | 67 (50.7) |  | 24 (18.1) |  | 17 (12.9) |  | 24 (18.1) |  | 132(100) |
| **Gestational age** |  | **< 0.005** |  | .101 |  | .103 |  | **< 0.005** |  |
| Preterm | 109 (33.3) |  | 75 (22.8) |  | 29 (8.9) |  | 115 (35) |  | 328(100) |
| Term | 171 (66.3) |  | 47 (18.2) |  | 32 (12.4) |  | 8 (3.1) |  | 258(100) |
| **Birth weight** |  | **< 0.005** |  | **< 0.005** |  | **< 0.005** |  | **< 0.005** |  |
| VLBW | 40 (30.5) |  | 11 (8.4) |  | 8 (6.1) |  | 72 (55) |  | 131(100) |
| LBW | 166 (55.5) |  | 64 (21.4) |  | 25 (8.4) |  | 44 (14.7) |  | 299(100) |
| NBW | 74 (47.4) |  | 47 (30.1) |  | 28 (18) |  | 7 (4.5) |  | 156(100) |
| **Diagnosis** |  | .485 |  | **.014** |  | **< 0.005** |  | **< 0.005** |  |
| EOS | 199 (47.9) |  | 76 (18.3) |  | 26 (6.3) |  | 114 (27.5) |  | 415(100) |
| LOS | 81 (47.4) |  | 46 (26.9) |  | 35 (20.5) |  | 9 (5.2) |  | 121(100) |
| **Mode of child birth** |  | **.021** |  | .296 |  | **.040** |  | **< 0.005** |  |
| SVD | 137 (52.7) |  | 51 (19.6) |  | 34 (13) |  | 38 (14.6) |  | 260(100) |
| C/S | 143 (43.9) |  | 71 (21.8) |  | 27 (8.3) |  | 85 (26) |  | 326(100) |
| **Resistant drugs** |  | .540 |  | .155 |  | .583 |  | .095 |  |
| >=5 | 16 (57.1) |  | 9 (32.1) |  | 1 (3.6) |  | 2 (7.1) |  | 28(100) |
| <5 | 12 (60) |  | 3 (15) |  | 0 |  | 5 (25) |  | 20(100) |
| **Length of hospital stay** |  | **< 0.005** |  | **< 0.005** |  | **.045** |  | **< 0.005** |  |
| <=5 days | 69 (26.3) |  | 88 (33.6) |  | 34 (13) |  | 71 (27.1) |  | 262(100) |
| >6 days | 211 (65.1) |  | 34 (10.5) |  | 27 (8.3) |  | 52 (16) |  | 324(100) |
| **Gram staining** |  | .052 |  | .321 |  | **< 0.005** |  | .488 |  |
| Positive | 5 (62.5) |  | 2 (25) |  | 1 (12.5) |  | 0 |  | 8(100) |
| Negative | 25 (51.7) |  | 12 (27.3) |  | 0 |  | 7 (15.9) |  | 44(100) |
| Not sent for culture | 62 (51.7) |  | 25 (20.8) |  | 3 (2.5) |  | 30 (25) |  | 120(100) |
| Culture negative | 188 (45.4) |  | 83 (20) |  | 57 (13.8) |  | 86 920.8) |  | 414(100) |
| **Modified treatment** |  | **< 0.005** |  | **< 0.005** |  | .310 |  | **< 0.005** |  |
| Yes | 125 (71.4) |  | 17 (9.7) |  | 16 (9.1) |  | 17 (9.7) |  | 175(100) |
| No | 155 (37.7) |  | 105 (25.5) |  | 45 (10.9) |  | 106 (25.8) |  | 411(100) |

VLBV=very low birth weight; LBW=low birth weight; NBW=normal birth weight; EOS=early onset sepsis; LOS=late onset sepsis; SVD=spontaneous vaginal delivery; C/S=caesarean section; p<0.05 considered statistically significant; Chi square test applied
