# Supplementary material for: Treatment outcomes, antibiotic use and its resistance pattern among neonatal sepsis patients attending Bahawal Victoria Hospital, Pakistan
Source: PLoS One. 2021 Jan 13;16(1):e0244866. doi: 10.1371/journal.pone.0244866 (PMC7806133; doi:10.1371/journal.pone.0244866)
Supplement: S3 File — (DOCX) [file pone.0244866.s003.docx]

**S3 File: Percentage of death with regard to modified treatment**

| **Modified treatment** | | **Death** | | | | **Total** | |
| --- | --- | --- | --- | --- | --- | --- | --- |
|  |  | **No** | | **Yes** | |  |  |
| Amikacin + ceftriaxone | 59 | | 14 | | 73 | |  |
| Vancomycin + ceftriaxone | 28 | | 1 | | 29 | |  |
| Vancomycin + meropenem | 9 | | 0 | | 9 | |  |
| Cefoperazone + sulbactam + ampicillin | 27 | | 2 | | 29 | |  |
| Cefoperazone + sulbactam + benzyl penicillin | 8 | | 0 | | 8 | |  |
| Imipenem + amikacin | 5 | | 0 | | 5 | |  |
| Imipenem + cefotaxime | 2 | | 0 | | 2 | |  |
| Imipenem + ceftriaxone | 5 | | 0 | | 5 | |  |
| Cefoperazone + sulbactam +amikacin | 17 | | 0 | | 17 | |  |
| Not modified due to any reason | 303 | | 106 | | 409 | |  |
| **Total** | | **463** | | **123** | | **586** | |
