# Supplementary material for: Treatment outcomes, antibiotic use and its resistance pattern among neonatal sepsis patients attending Bahawal Victoria Hospital, Pakistan
Source: PLoS One. 2021 Jan 13;16(1):e0244866. doi: 10.1371/journal.pone.0244866 (PMC7806133; doi:10.1371/journal.pone.0244866)
Supplement: S4 File — (DOCX) [file pone.0244866.s004.docx]

**S4 File: Number of deaths with regard to culture status**

| **Culture status** | | **Death** | | | | **Total** | |
| --- | --- | --- | --- | --- | --- | --- | --- |
|  |  | **No** | | **Yes** | |  |  |
| Positive | 45 | | 7 | | 52 | |  |
| Negative | 328 | | 86 | | 414 | |  |
| Specimen not sent for culture | 90 | | 30 | | 120 | |  |
| **Total** | | **463** | | **123** | | **586** | |
